# Apple whole genome sequences: recent advances and new prospects

**DOI:** 10.1038/s41438-019-0141-7

**Published:** 2019-04-05

**Authors:** Cameron P. Peace, Luca Bianco, Michela Troggio, Eric van de Weg, Nicholas P. Howard, Amandine Cornille, Charles-Eric Durel, Sean Myles, Zoë Migicovsky, Robert J. Schaffer, Evelyne Costes, Gennaro Fazio, Hisayo Yamane, Steve van Nocker, Chris Gottschalk, Fabrizio Costa, David Chagné, Xinzhong Zhang, Andrea Patocchi, Susan E. Gardiner, Craig Hardner, Satish Kumar, Francois Laurens, Etienne Bucher, Dorrie Main, Sook Jung, Stijn Vanderzande

**Affiliations:** 1 Department of Horticulture, Washington State University, Pullman, WA 99164 USA; 2 Computational Biology, Fondazione Edmund Mach, San Michele all’Adige, TN 38010 Italy; 3 Department of Genomics and Biology of Fruit Crops, Fondazione Edmund Mach, San Michele all’Adige, TN 38010 Italy; 4 Plant Breeding, Wageningen University and Research, Wageningen, 6708PB The Netherlands; 5 Department of Horticultural Science, University of Minnesota, St. Paul, MN 55108 USA; 6 Institut für Biologie und Umweltwissenschaften, Carl von Ossietzky Universität, 26129 Oldenburg, Germany; 7 GQE – Le Moulon, Institut National de la Recherche Agronomique, University of Paris-Sud, CNRS, AgroParisTech, Université Paris-Saclay, 91190 Gif-sur-Yvette, France; 8 Institut National de la Recherche Agronomique, Institut de Recherche en Horticulture et Semences, UMR 1345, 49071 Beaucouzé, France; 9 Department of Plant, Food and Environmental Sciences, Faculty of Agriculture, Dalhousie University, Truro, NS B2N 5E3 Canada; 10 The New Zealand Institute for Plant and Food Research Ltd, Motueka, 7198 New Zealand; 11 School of Biological Sciences, University of Auckland, Auckland, 1142 New Zealand; 12 AGAP, INRA, CIRAD, Montpellier SupAgro, University of Montpellier, Montpellier, France; 13 Plant Genetic Resources Unit, USDA ARS, Geneva, NY 14456 USA; 14 Laboratory of Pomology, Graduate School of Agriculture, Kyoto University, Kyoto, 606-8502 Japan; 15 Department of Horticulture, Michigan State University, East Lansing, MI 48824 USA; 16 The New Zealand Institute for Plant and Food Research Ltd (Plant & Food Research), Palmerston North Research Centre, Palmerston North, 4474 New Zealand; 17 College of Horticulture, China Agricultural University, 100193 Beijing, China; 18 Agroscope, 8820 Wädenswil, Switzerland; 19 Queensland Alliance of Agriculture and Food Innovation, University of Queensland, St Lucia, 4072 Australia; 20 New Cultivar Innovation, Plant and Food Research, Havelock North, 4130 New Zealand; 21 Agroscope, 1260 Changins, Switzerland

**Keywords:** Genomics, Agricultural genetics, Plant breeding, Functional genomics, Genome

## Abstract

In 2010, a major scientific milestone was achieved for tree fruit crops: publication of the first draft whole genome sequence (WGS) for apple (*Malus domestica*). This WGS, v1.0, was valuable as the initial reference for sequence information, fine mapping, gene discovery, variant discovery, and tool development. A new, high quality apple WGS, GDDH13 v1.1, was released in 2017 and now serves as the reference genome for apple. Over the past decade, these apple WGSs have had an enormous impact on our understanding of apple biological functioning, trait physiology and inheritance, leading to practical applications for improving this highly valued crop. Causal gene identities for phenotypes of fundamental and practical interest can today be discovered much more rapidly. Genome-wide polymorphisms at high genetic resolution are screened efficiently over hundreds to thousands of individuals with new insights into genetic relationships and pedigrees. High-density genetic maps are constructed efficiently and quantitative trait loci for valuable traits are readily associated with positional candidate genes and/or converted into diagnostic tests for breeders. We understand the species, geographical, and genomic origins of domesticated apple more precisely, as well as its relationship to wild relatives. The WGS has turbo-charged application of these classical research steps to crop improvement and drives innovative methods to achieve more durable, environmentally sound, productive, and consumer-desirable apple production. This review includes examples of basic and practical breakthroughs and challenges in using the apple WGSs. Recommendations for “what’s next” focus on necessary upgrades to the genome sequence data pool, as well as for use of the data, to reach new frontiers in genomics-based scientific understanding of apple.

## Genetics: Apple genome sequencing begins to bear fruit

Almost 10 years since the first draft of the apple genome was published, the insights it has afforded are being used to improve crops, while next generation DNA sequencing is enabling the breeding value of individual plants to be more rapidly assessed. In this review, Cameron Peace at Washington State University in Pullman, US, and colleagues describe the impact whole genome sequencing of the Golden Delicious apple has had on our understanding of how cultivated apples evolved, and the genomic regions controlling fruit firmness and flavor, tree growth dynamics, responses to water and nutrient availability, and other such traits. These early discoveries have also paved the way for trait-predictive tests which should further accelerate the breeding of improved apple trees, and epigenetic studies to better understand how environmental factors trigger heritable changes in apple characteristics.

## Introduction

In 2010, a major scientific milestone was achieved: publication of the first reference whole genome sequence (WGS) for cultivated apple (*Malus domestica*). Reported by Velasco and co-authors^1^, the first apple WGS resulted from the collaborative efforts of scientists from 14 institutions in five countries. Apple was the tenth plant genome to be sequenced, after *Arabidopsis*, rice, poplar, grape, papaya, sorghum, cucumber, maize, and soybean^2^. A WGS ideally provides the identity of each nucleotide, in order for each chromosome, in a genome. As this sequence is largely consistent across the nuclei of all living cells of an organism, the WGS fundamentally represents an organism’s internal genetic instructions. “Golden Delicious” was chosen to represent the crop from this genomic perspective because it is a century-old cultivar that remains popular in commercial production across the globe^3^ and is also highly prominent in the recent ancestry of a large proportion of modern apple cultivars^4–7^. Similar to other apple cultivars, “Golden Delicious” is highly heterozygous (3.2 single nucleotide polymorphisms [SNPs] per 1000 bp and *π* = 0.0032 ± 0.0032^1^. Sequence fragments were compiled in silico into overlapping, contiguous segments (i.e., contigs) then anchored to their most likely positions along the 17 chromosomes of apple. The anchoring used a six-family (*n* = 720 offspring total) genetic map with 1643 genetic markers, mostly SNPs from the “Golden Delicious” genome itself^1^. With this approach, contigs spanning 75% of the genome were determined to be correctly oriented, thus leaving 25% of contigs with “uncertain orientation”. This apple WGS covered approximately 81% of the genome, as the average length of assembly was 604 Mb compared to the estimated genome size of 742 Mb^1^. The N50 for this original WGS was only 16.7 kb (i.e., half the assembly was composed of contigs ≥16.7 kb in length). This WGS was described as a “high-quality draft”—not fully complete, but well worth releasing to the scientific community.

Excitement surrounded publication of the apple WGS: it promised to reveal how apple as a species and a crop arose, to help elucidate how apple trees and organs function, and to provide “a tool to initiate a new era” in apple genetic improvement via breeding. Key basic discoveries reported by Velasco and co-authors^1^ were: (1) the 17 chromosomes of the apple genome are monophyletically derived from a relatively recent genome-wide duplication of an ancestral 9-chromosome Rosaceae ancestor, giving a particular pattern of chromosomal homologies; (2) the gene pool of cultivated *M. domestica* was formed primarily from the wild species *M. sieversii*; (3) the distinctive pome fruit of apple and its close relatives appears to be the result of expansion of a *MADS-box* fruit-development gene family; and (4) gene families involved in metabolism of sorbitol, the primary transport molecule in Rosaceae for photosynthesis-derived carbohydrates, are also expanded in the apple genome compared to non-Rosaceae genomes. Although it complicated genome assembly, the high heterozygosity of the sequenced cultivar was embraced intentionally for the “practical goal […] to accelerate the breeding of this economically important perennial crop species”^1^. The authors reported specifically on the genomic positioning of candidate genes for apple production and consumer traits. Allelic differences in the form of SNPs between the two parental homologs of each “Golden Delicious” chromosome were compiled to accelerate cultivar development during breeding. The publication also highlighted the availability of sequences and revealed positions for nearly all genes of apple, to enable genome-wide research of gene functions and their association with traits of value.

A new, very high quality apple WGS has recently succeeded this first apple WGS and now serves as the reference genome for basic and practical scientific advances. A doubled-haploid derivative of “Golden Delicious” known as GDDH13 was sequenced to unprecedented depth with short and long read sequencers and assembled de novo^8^ in combination with optical maps. The total length of this assembly was 643 Mb, with 42,140 annotated protein-coding genes. The publication investigated the highly repetitive “dark matter” of the genome—transposable elements (TEs), which represented 60% of the genome in the final assembly and provided insights into apple genome evolution^8^. Just prior to the GDDH13 WGS publication, an improved apple WGS of heterozygous “Golden Delicious” was reported that increased the N50 of contigs from the original WGS by seven-fold to 112 kb^9^. The GDDH13 N50 was massively higher again, at 5.558 Mb^8^. At present, when referring to the apple WGS, researchers almost always mean the GDDH13 reference genome.

As promised, the apple WGS has indeed served as a springboard to further scientific advances by the research community. The original paper has been cited almost 1000 times in the eight years since publication, according to *Nature Genetics*. The following series of vignettes is a non-exhaustive collection describing how the apple WGS (the original as well as the new reference genome, GDDH13) has facilitated fundamental discoveries across the world about *M. domestica* and served as a resource for practical applications in apple crop improvement. Topics cover both physiological and genetics advances, separately or intertwined. Almost a decade of research has identified challenges and limitations to scientific aspirations in using the apple WGS, some of which have successfully been overcome with the GDDH13 WGS^8^ or are otherwise being creatively addressed. We highlight opportunities to reach new heights in genomics-based scientific advancement of apple.

## Genome-wide SNP marker development: capturing allelic variation

The publication of the first high-quality apple reference genome^1^ and following updates^8^ have enabled whole genome investigations for this fruit crop. Reference-based variation discovery approaches compare re-sequencing information to the reference genome assemblies, in order to identify differences between them. Several different approaches exist for this analysis. The first, and conceptually most logical, is high-coverage whole genome sequencing where one or more plants are independently resequenced and compared to the reference through de novo or reference-based assembly. This approach has the advantage of giving potential access to the whole variation (SNPs, insertions, deletions, transversions, copy number variants, etc.) of the resequenced plants, but has the drawback of high sequencing costs (long reads and chromosome scale scaffolding techniques are needed), requires quite complex data analysis, and demands high-performance computing platforms. A simpler approach would be to focus only on SNPs and small insertions/deletions (indels). In this case, sequences (mostly short Illumina reads) can be aligned directly to the reference genome to identify variants. This approach has been followed in many studies both on a whole genome level and by making use of restricted genome libraries such as in genotyping-by-sequencing (GBS).

Three SNP genotyping arrays have been developed for apple. An initial Illumina 8K SNP array was developed from whole-genome resequencing of 27 cultivars at low sequencing coverage^10^. For the Illumina 20K SNP array, the discovery panel consisted of 14 major founders of European breeding programs at relatively high sequencing coverage^11^. Most recently, a total of 67 resequenced accessions were studied to build the largest SNP genotyping array yet available for a fruit tree, the Affymetrix Apple480K^12^. The advantages of these genome-scanning tools include their availability to the whole research community, ability to assay a consistent set of loci each time they are used, and rapid production of results that are relatively easy to interpret for hundreds to thousands of individuals. A drawback is ascertainment bias, because the SNPs included in each array were obtained from discovery panels of particular accessions that were purposely chosen to represent modern breeding germplasm of *M*. *domestica* scion cultivars. Therefore, these arrays might not suit some specific research needs where rare or other *Malus* species-specific variants are important.

The GBS approach, in contrast, does not use a fixed set of SNPs—with corresponding advantages and disadvantages. GBS resources have been used to generate a saturated genetic linkage map of the apple genome^13^ and for genome-wide association study (GWAS) of fruit quality, harvest date, scab resistance, and leaf shape^14–17^. However, GBS generates a sparse genotype matrix due to uneven sequence coverage across individuals screened from the germplasm and across loci. SNP-calling pipelines for GBS designed specifically for highly heterozygous and polyploid species that incorporate haplotype phasing and imputation promise to significantly enhance the utility of GBS^18–21^. Aggregation of sequence information across each haploblock^22^ might be another strategy to improve genome-wide genotyping efficiency. Imputing haplotypes for haploblocks and determining their contribution to QTLs is already possible using dense SNP array data, even in polyploids such as rose^23^.

Future investigations in this area should: (i) expand the concept of a single genome for apple towards that of the pan-genome concept, whereby the genomic variation of the entire crop is captured, annotated, and made available to the research community, through tools capable of highlighting *M*. *domestica* intraspecific sequence differences and associating them with phenotypic effects; and (ii) incorporate wild relatives of apple to enable scouting for the genomic variants responsible for valuable attributes for introgression into apple cultivar breeding programs.

### Genetic linkage mapping: increased accuracy for the recombination context of the genome

Genetic linkage maps are employed in marker-trait discovery studies, map-based cloning of genes and the assembly and/or validation of genome sequences, as well as in genetic diversity and relatedness studies that make use of marker haplotypes^7,24^. A genetic linkage map presents an estimated order and recombination distance of loci along each chromosome composing the genome of a species. Genetic maps constructed to date have mostly resulted from co-segregation analyses of DNA-based markers in full-sib families. Marker ordering can also be based on high-quality reference genome sequences, such as that for peach^25^. This latter approach can enhance downstream genetic studies by reducing effort required for map construction and by enhancing the quality of the genotypic data, because small regions of double recombination are likely to arise from genotyping errors and spurious genotypes can be easily traced, examined and curated where necessary^26^. Linkage maps generated de novo might still be useful for validation and improvement of already available whole genome sequences, for ordering de novo assemblies of wild *Malus* species, and for identifying and validating genomic re-arrangements and large indels.

The original “Golden Delicious” WGS^1^ did not allow adequate genome-wide ordering, but subsequent research building on it has helped. From 11 to 36% of markers used in genetic linkage map development showed mismatches between genetic and WGS v1.0 physical positions^8,27–30^. Nevertheless, physical position information was successfully exploited in designing the 8K, 20K, and 480K apple SNP arrays, where sets of SNP markers were developed for narrow physical bins^10–12^. This design supported the use of haploblocks of aggregated SNPs in genetic analyses, facilitating the development of a new approach for the construction of high quality linkage maps in outbreeding species^7,30,31^. The most comprehensive linkage map in apple to date was developed in this manner, the iGL map represents more than 3000 meioses derived from 21 full-sib families and 15,417 SNP markers^30^.

**Fig. 1:**
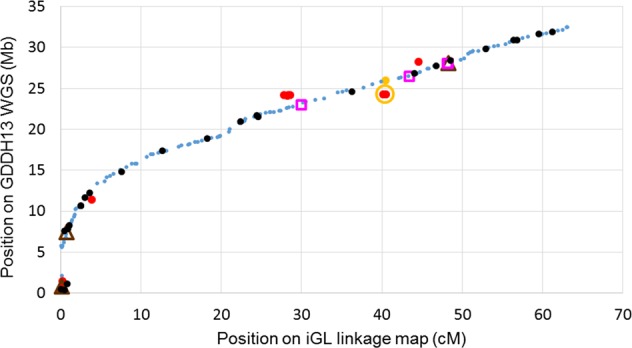
Haploblock markers of LG 1 plotted for position on the GDDH13 reference WGS compared to that on the iGL genetic linkage map.  = matching order.  = matching after shuffling within genetic bins. ,  = the two positions of markers with two full hits on chromosome 1 whereas their cluster plots showed them to be true single locus markers; only the former position matched the iGL map (one haploblock with five SNPs jointly representing two continuous stretches of 2.9 kb with 99.8% sequence similarity).  = cM-Mb mismatches that could not be resolved through shuffling on the iGL map and for which calls and hit-qualifications were appropriate; they matched with a 0.8 to 5.5 Mb shift along the chromosome (12 haploblocks with 35 SNPs). ,  = no hit on chromosome 1 but on another chromosome (four haploblocks with seven SNPs) or no hit at all (three haploblocks with four SNPs), respectively; their physical coordinates were estimated based on the nearest matching flanking markers on the iGL map

Although the more recent GDDH13 WGS is an improvement over the original WGS^8,30^, inconsistencies between genetic and physical positions still exist. For example, the gene *Mal d 1.06* was mapped genetically to linkage group (LG) 6 by means of a gene-specific sequence characterized amplified region (SCAR) marker, while the marker’s primer sequences were retrieved within a known *Mal d 1* gene family cluster on GDDH13 chromosome 13^32^. In addition, 127 (0.8%) of the 15,417 SNP markers of the iGL map could not be retrieved, suggesting the presence of indels or missing chromosome fragments. Current detailed analyses of LG 1 (Fig. 1) indicate that the iGL map and the GDDH13 v1.1 sequence are highly compatible in most positions. However, the iGL map located two GDDH13 v1.1 regions where the assembly might need further improvement, namely the first 5.8 Mb of the chromosome (where the centromere is located) and the 22.3–28.4 Mb region (Fig. 1), where LG-chromosome and cM-Mb mismatches, unlikely duplications, and un-retrieved iGL markers were noted (Fig. 1). Missing sequence information from the apple WGS was reported where the promoter of *MdCPD1* (chromosome 1, 26.2 Mb) could not be retrieved^33^, and as well as a similar case where cloned *MdVQ* genes that initially had been identified in the “Golden Delicious” sequence could not be recovered from the GDDH13 v1.1 sequence^34^. Hence, use of the GDDH13 WGS in marker ordering, as well as for other uses, is not yet straightforward. In the near future, it might be useful to extend the co-linearity between highly curated genetic maps and the GDDH13 WGS to the entire genome and tag suspicious regions in genome browsers.

## Apple domestication: a story of gene flow

The domestication history of apple started in Central Asia, spreading towards the West in Europe and beyond, assisted by propagation of interesting genotypes by grafting^35^. Recent investigations using population genetic approaches with microsatellite markers have demonstrated that wild-to-crop gene flow played a major role in the evolutionary history of the cultivated apple^36^. Reciprocally, crop-to-wild hybridizations have spread alleles from the cultivated gene pool to populations of wild *Malus* species^37–39^, with possible negative fitness consequences for the wild gene pool^39^. Such hybridizations, followed by successful establishment of wild beneficial alleles into domesticated populations and vice versa, can occasionally trigger adaptation. For example, adaptive introgression from highland teosintes contributed to maize highland adaptation^40^. However, so far no study has investigated the adaptive consequences of crop-wild introgressions in apple and their genomic bases. Questions of particular relevance include: What is the genomic architecture of introgressions (number of genes involved, locations, and nature of the genes and their alleles) between the cultivated apple and its wild apple relatives? Have these introgressions been selected upon during apple evolution or domestication, and what form do they take (e.g., amino-acid substitution, gene duplications, gene gains, gene losses)? More generally, are these introgressions adaptive? Resolving the last question, i.e., demonstrating the fitness advantages of the introgressed allele and its associated attribute for the recipient population, would be time-consuming or even infeasible in apple because its long generation time would render field experiments challenging^35,41^. Fortunately, next generation sequencing technologies can be used to elucidate the first two questions by detecting genomic variation underlying adaptation of wild and cultivated apple populations to their biotic and abiotic environments and fruit-related traits.

Numerous publications involving genome-wide data are accumulating for fruit crop perennials^8,42–54^, but they still lag behind those for other crop plant systems^41,55,56^. In apple, the few studies available have helped to gain initial insights into the genomic basis of apple evolution and domestication^8,35,55^. For example, a GWAS that included both the cultivated apple and, for the first time, several wild apple species (in particular *M. sieversii*, *M. orientalis* Uglitz., and *M. sylvestris* Mill.) revealed genomic regions determining fruit firmness and flavor and explained how these regions have been affected by selection during domestication^57^. This study supported a model of apple fruit size evolution comprising two major events, with one occurring prior to domestication and the other during domestication. Similarly, but without the use of genome-wide data, a work by Yao and co-authors^58^ showed that a microRNA whose expression is associated with fruit size was fixed in cultivated apple and in their wild progenitors with large fruit, indicating that selection for fruit size was initiated before apple domestication. Next generation sequencing technologies have also been used to identify the genes underlying resistance to pathogens, such as blue mold (*Penicillium expansum*) infection in *M. sieversii* compared to *M. domestica*^59^ and to identify trait loci controlling resistance to this pathogen in *M. sieversii*^60^. Genomic analyses therefore suggest that apple differs from annual crop models, such as maize^40^, for which most key agronomic traits were likely selected after domestication^61^. However, so far, these genomic studies of apple have not integrated the estimation of the timing of selection associated with such pre-existing or post-domestication adaptation to a new environment (e.g., climate, pathogens, and human) and have not tested whether such genomic regions are the result of crop-wild introgressions following apple domestication.

Wider sampling of the wild apple relatives across their natural Eurasian distributions, in Central Asia, the Caucasus, and Europe (*M. sieversii*, *M. orientalis*, and *M. sylvestris*, respectively), as well as of local *M. domestica* cultivars from the same regions, and more ancestral species (*M. baccata*, *M. floribunda*, *M. florentina*, *M. niedzwetzkyana*), will help resolve the timing of adaptation and the role of gene flow in apple evolution and domestication. Indeed, sequential temporal wild-crop introgressions have been previously detected using microsatellite markers from the East (e.g., from *M. orientalis*) to the West (e.g., from *M. sylvestris*)^35^. Development of new population genomic inference methods that allow estimates of the dynamics of gene flow and positive selection between populations^62–65^, combined with a comprehensive sampling of both local wild populations and cultivars, now offer clear opportunities to explore the timing, genomic architecture, and regimes of selection acting in the introgressed regions in wild and cultivated apple and will help clarify the role of gene flow in this fascinating perennial model system.

## Characterization of germplasm collections: diversity, origins, and worldwide pedigree connections

More than 10,000 apple cultivars have been described worldwide^66^. Thousands of these cultivars are curated in large national repositories, in addition to those managed by private institutes or associations and active amateur networks, especially in Europe and the U.S. Until the end of twentieth century, characterization of each cultivar was mostly phenotypic, using pomological traits, phenology, fruit sensory quality, etc. Genetic characterization with simple sequence repeat (SSR) markers has been performed intensively in the last 10–15 years, allowing identity checking and designations of synonym groups (containing synonymous cultivars and sports), assessment of the genetic structure at the collection or multi-collection level and preliminary parentage inferences among cultivars (e.g., see ref. ^67–69^).

The apple 20K and 480K SNP arrays^11,12^, enabled by the apple WGSs^1,8^, have been widely used to genotype numerous apple cultivars from various, mostly European, germplasm collections. The high-resolution genetic information developed allows unprecedented parentage inferences, revealing a very dense and unsuspected kinship network, with key cultivars at the top of the European pedigree including “Reinette Franche”, “Margil”, and “Alexander”^70^. Parentage of numerous well-known cultivars such as “White Transparent”, “Ribston Pippin”, and “Braeburn” has been elucidated. Recent extension of the analysis to the wild apple gene pool included resequencing of more than 110 domesticated and wild accessions and resulted in a comprehensive model of apple speciation and domestication along the Silk Road, identifying introgressions from the European *M. sylvestris* and signatures of selective sweeps underlying loci influencing important fruit quality traits, notably fruit size^57^.

Numerous short-term and long-term research opportunities beckon. In the short term, evaluating germplasm for allelic variation at breeding-relevant trait loci will help reveal the practical value inherent in collections. Additional outcomes are expected from pedigree reconstruction to infer the European ancestors of numerous heritage U.S. cultivars^71^ and to better decipher pedigree relationships across Northern and Southern Europe genepools. Calculation of haplotype sharing along and beyond the inferred pedigrees will make it possible to identify and visualize portions of the genome shared (or not) between two or more cultivars, with major implications for fostering crossing and “Breeding by Design” approaches^72^. Identification of signatures of empirical selection across and beyond the inferred pedigree should be sought to further decipher genomic regions and corresponding genes under selection in the past, with expected insights into the history of apple breeding over previous centuries. Also, effective population sizes in both germplasm and breeding populations, accounting for pedigree information, should be determined, with implications for management of genetic diversity and filling gaps in collections. In the longer term, future research might address the exploration of core and pan-genomes, taking advantage of extensive resequencing of germplasm collections (including wild *Malus* species), and exploring the putative role of copy number variants (CNVs) in the wide phenotypic variation of apple and related *Malus* species. The location of genomic regions with haplotypes introgressed from wild *Malus* species (especially *M. sylvestris* and *M. orientalis*) in the domesticated pool should help to assess their impact on adaptive and agronomic value. Another exciting domain is the exploration of epigenetic modifications, taking advantage of bisulfite resequencing or other approaches, especially in very old or widely-dispersed cultivars that have accumulated hundreds of years of modifications, to bring insights into aging and adaptation processes.

## Methods for genetic dissection of traits: enhanced Genome-Wide Association Study and Pedigree-based analysis (PBA)

Genetic mapping of trait loci in apple has commonly been performed by interval mapping using bi-parental families. Although the underlying genetic models are simplified in such population structure, genomics-assisted apple improvement will be severely limited if bi-parental families are the primary material for detecting and characterizing apple Mendelian trait loci (MTLs) and quantitative trait loci (QTLs), for three main reasons. Firstly, only the genetic variation segregating within the parents of a cross can be queried, and thus only a small fraction of the apple’s tremendous genetic diversity is examined when only two parents are involved. Secondly, marker-trait associations identified in one family are often not transferable to others, and this lack of transferability is a hindrance to apple breeders eager to exploit marker-trait associations. Finally, the limited number of recombination events captured in bi-parental crosses results in poor mapping resolution, such that gene-level mapping resolution is unlikely and the identification of causal alleles becomes largely guesswork. Some of these limitations can be overcome by using a PBA approach to discover QTLs^73–75^. In this approach, multiple (often smaller) families that are pedigree-connected through immediate and distant progenitors are jointly analyzed. The families are chosen to represent important breeding parents and their alleles, preferably segregating in multiple genetic backgrounds to enable the simultaneous identification and validation of QTLs in a breeding-relevant context^76^. This approach has been successfully used in multi-institution international projects such as RosBREED^77,78^ and FruitBreedomics^79,80^, as well as in national projects^81^. For QTLs only segregating in certain small families, QTLs might not be detected, their intervals can remain large, and estimates of allele effects can be inaccurate—although these are challenges facing any QTL detection method where allelic contrasts are not well represented in the investigated germplasm.

The release of the apple WGS has been a crucial step in enabling an alternative genetic mapping method, GWAS. GWAS detects genotype–phenotype associations in diverse populations that capture all historical recombination events within the sample^82^. Thus, GWAS in large, diverse populations overcomes the three limitations of bi-parental linkage mapping outlined above: GWAS captures more diversity, results in improved transferability, and enables higher mapping resolution.

DNA sequence reads generated from next-generation DNA sequencing (NGS) must be aligned to a reference genome before performing GWAS. The effectiveness and accuracy of this alignment relies heavily on the completeness and quality of the reference genome. As described in the previous section on genetic linkage mapping, a substantial proportion of genome-wide markers were assigned to the wrong chromosomes in the apple reference genome described by Velasco and co-workers^1^. Thus, the current improvements in genome assembly^8^ should dramatically enhance the success of the very first step in the NGS genotyping process, resulting in higher marker densities, higher quality genotype calls, and ultimately more powerful GWAS.

Genome mis-assembly also makes it difficult to draw conclusions about the genetic architecture of a trait following GWAS. For example, a recent GWAS for apple fruit color identified the well-studied color locus, *MYB1*, on chromosome 9, but also identified a marker on chromosome 17 significantly associated with color. Can we safely assume then that there are two large-effect loci controlling apple color? The answer in this case is no—only a single locus is involved. The chromosome 17 marker’s position was incorrect, and it actually belonged on its homoeologous chromosome 9 with the other significant markers in and around *MYB1*^14^. Similarly, incorrect marker order also perturbs widely reported metrics of genomic diversity such as the decay of linkage disequilibrium (LD), which will appear to be more rapid when marker order is muddled in incorrect genome assemblies.

Despite some limitations due to genome assembly errors, GWAS using the original apple reference genome^1^ has proven an effective alternative to traditional interval mapping approaches. For example, GWAS has identified large-effect loci for firmness, firmness retention, flowering and harvest dates, internal flesh browning and fruit splitting that remained undiscovered, despite numerous interval mapping studies^14,17,83,84^. Such discoveries are leading to improvements in the efficiency of apple breeding. For example, a firmness marker in the *NAC18.1* transcription factor discovered via GWAS^14^ is currently being applied by Canadian apple breeders.

The release of the apple WGS has reinforced use of the above-mentioned PBA approach by facilitating high-density genome-wide genotyping as well as exploiting haploblocking. The former empowers FlexQTL™’s functionality on estimating genome-wide breeding values, thereby allowing to combine the contributions of statistically distinct QTLs with minor effects associated with genetic bins. The use of haploblocks dramatically reduces computation time with little loss of segregation information^24^. In this respect, FlexQTL^TM^ based PBA analysis has been ahead of GWAS and genomic prediction approaches when pedigree-connected families are in use.

## Physiological roles of apple genes: discovering what the genes do

Understanding gene function in any plant is still a challenge, and this is magnified in a slow-growing perennial such as apple. However, the plummeting cost of sequencing has changed the way researchers can interrogate the physiological roles of apple genes. Reduced costs initially allowed researchers to move from mass sequencing of ESTs^85,86^ to whole genomes^1,8,9^ and on to crops, rather than model species, to characterize gene function. The apple WGS now provides the gene and its genomic environment: the promoter, intron-exon structure, flanking sequences, and neighboring genes. Gene expression can be measured by high-throughput sequencing, without the need to choose individual genes for expression studies. Next, rapid resequencing and alignment of diverse genomes can be used to identify allelic differences that lead to the desired physiological effects to enrich for in breeding programs^57^.

Knowledge of gene function starts with the sequence of the gene, which can be obtained from the WGS with a simple bioinformatic search. Function can be further inferred using knowledge about where the gene is expressed, when and where the corresponding protein is produced, and how the protein interacts with other proteins and compounds to create an effect. Each of these steps requires an increasing amount of research effort. However, the most accurate test is to knock the gene out and/or overexpress the gene to verify its function. This gene perturbation can be performed transiently with a virus-induced gene silencing approach (e.g., see ref. ^87^) or via a stable transformation (see ref. ^88–90^). The creation of a stable transgenic apple plant was described more than 30 years ago^91^, but transformation of this crop is still not trivial, restricting the numbers of genes that can be realistically evaluated. The use of model species to rapidly assess apple gene function is one effective method to increase the numbers of genes that can be scrutinized. This pre-functional testing has been demonstrated for the case of genes conferring red coloration^92^.

With knowledge of the specific genes involved in controlling traits of interest, the possibility opens to use technologies that overcome certain biological limitations of breeding. There are clear breeding targets in apple to generate consumer-attractive apples that are produced in a sustainable, low-impact production line^93,94^. While transgenic plants are an option in some countries^95^, breeding programs in more restrictive environments must rely on natural variation to generate new cultivars. New technologies such as CRISPR have been used in apple to create mutations in genes. This application of gene editing does not trigger regulation for genetically modified organisms (GMOs) in the US and in some other countries, but it will be subject to the GMO regulatory process in the EU, even if the mutation is identical to one that has occurred naturally in another variety^96^. To accommodate this new technology, experts are challenging the European Commission to review the decision of the European Court of Justice in July 2018^97^.

Future research to further understand the physiological roles of apple genes should include the following. Data integration: We have moved from a point where data was hard to generate and was typically over-analyzed to a point where data is easy to generate and often under-analyzed. Large data sets are often treated as a separate entity, with little integration of information from prior publications. There is a massive opportunity to address the way that data is integrated. This involves not only data from apple experiments, but across all plants. Faster gene function analysis: Tools and approaches for higher-throughput gene investigation are needed to further understand how individual apple genes are acting. This will allow more informed choices of genes to focus on as well as alleles to select for in breeding and as CRISPR targets. Focus on the proteins: The way we analyze nucleotides (DNA/RNA) has had a massive revolution in the last 20 years. We need a corresponding revolution in protein-sequencing technologies that are sensitive, high-throughput, and affordable. Once we have this advance, we can begin to dissect the functional biology of apple.

## Tree architecture genes and genetics: a growing understanding from the genome

Apple tree architecture is highly organized throughout ontogeny and at different scales. Growth, branching and flowering are the main processes giving rise to plant architecture and are key determinants of productivity. Genetic and genomic study of these traits is hence of great relevance for breeding. Progress in understanding the genetics of architectural traits is limited by the large size and long life of apple trees, which makes it difficult to assess phenotypes on large numbers of individuals. Nevertheless, significant advances have been obtained in genetics studies performed on architectural traits in apple and on the underlying physiological and molecular mechanisms. Early genetic studies underlined the genetic variation in tree forms and architectures of apple^98^. Heritability was estimated for basic morphological traits such as tree height or trunk diameter^99,100^ and QTLs mapped for branching habit evaluated qualitatively^101^ or for global geometric traits such as trunk height and base diameter^102^. The dissection of architecture into elementary traits led to many QTL mapping studies with bi-parental segregating families, the results of which have suggested complex genetic control^103,104^.

The apple WGS has supported further advances in understanding the genes and genetic variation controlling apple tree architecture. The WGS has allowed the search for candidate genes in QTL zones, possibly combined with the characterization of mutants or sports that display interesting phenotype^105^. In apple, the discovery of natural mutants exhibiting a columnar compact growth habit^106^ is certainly the most well-known, as this habit has been considered suitable for high density orchards. The columnar habit of “Wijcik” was mapped at a locus on LG 10 named *Columnar* (*Co*)^107^. QTLs have been mapped for trunk and branch geometry (length and base diameter) and for sylleptic branching in progenies derived from a columnar parent^108,109^, most of them colocalized with *Co*, suggesting pleiotropic effects of *Co*. The WGS assisted in the fine mapping of *Co*, through the directed development of genetic markers^110–112^, the identification of BAC clones that jointly covered the *Co* region^113–115^ and the directed Sanger sequencing of fragments that connected the BAC-derived contigs^113,115^ or assisted alignment of Illumina whole genome sequencing data to the *Co* region^114^ and finally the identification of the causal gene. The columnar phenotype of the dominant allele of the *Co* locus has been shown to be due to an insertion of a *Ty3/Gypsy* retrotransposon that likely upregulates a nearby gene encoding a 2OG-Fe(II) oxygenase^113–116^.

The WGS has greatly facilitated transcriptomic studies as it has allowed the annotation of differentially expressed genes involved in tree architecture. The columnar mutation was associated with the differential expression of a vast number of genes regulated in the shoot apical meristem^117^. Another example is the regulation of tree architecture by dwarfing rootstock, which has recently been shown to involve unbalanced carbohydrate allocation and downregulation of auxin influx transporters *MdAUX1* and *MdLAX2*^118^.

The WGS enables searching for all genes in a given family, as proposed for screening the *IGT* family genes, that have been assumed to play a crucial role in apple architecture^119^. Four *IGT* family members (*MdoTAC1a*, *MdoTAC1b*, *MdoLAZY1*, and *MdoLAZY2*) have been identified in the apple WGS and characterized in four apple cultivars with contrasting architecture. This study^119^ revealed mutated sites in promoters and differential expression of these genes in all tissues and organs evaluated, in the four cultivars.

All these studies demonstrate the enormous potential of ongoing genomic investigations to aid in the development of an understanding of the genetic control of modified tree architecture. Results suggest that many of the key genetic pathways are functionally conserved across species^120^. The logistics of phenotyping large populations of trees with diverse architecture is currently a major bottleneck. If this challenge could be overcome, genomic studies could readily exploit genetically variable multi-family populations composed of related individuals, such as breeding pedigrees or unrelated accessions in germplasm collections. From this perspective, technologies based on terrestrial Lidar and imaging are promising emerging tools^121^. We can expect that the tremendous genetics advances achieved during the last decade and which are ongoing through the development of high-throughput genotyping, phenotyping, and genomic studies will open new avenues for describing plant architecture and exploring the mechanisms of its genetic and molecular physiological control.

## Water and nutrient use efficiency genes and genetics: absorbing new genomics advances

Apple WGSs have helped identify genes and genetic factors involved in the complex and valuable traits of apple tree responses to water and nutrient availability. Water use efficiency (WUE) determines production capacity in regions subject to drought and low water availability and for which advances in knowledge have been made using genomic approaches. WUE is defined by the amount of photosynthesized carbon per units of transpired water and is commonly measured seasonally (units of dry matter seasonal growth per unit of water) or by measuring CO_2_, O_2_, and H_2_O fluxes of tree canopies during short periods^122^. In combination with phytohormones and root morphology, WUE is thought to be associated with drought tolerance in apple^123,124^. Phenotypic diversity for physiological and morphological components of WUE was reported in domesticated apple and related wild species and several genes responding to water deficit have been described in apple roots^125^. Mineral nutrients at the correct concentration play pivotal roles in all physiological functions of apple trees, where a deficiency can lead to fruit disorders such as bitter pit^126^, while an excess can lead to toxicity disorders such as boron shoot die back^127^. A perturbation of nutrient-related physiological functions will hence have repercussions on horticultural performance of the whole tree as on apple fruit quality^126,128–131^.

Use of the apple WGS v1.0 advanced the genetic dissection of WUE in QTL and gene-based studies. QTLs were identified for proxies of transpiration traits derived from airborne images collected on a “Starkrimson” × “Granny Smith” family that was genotyped with SSR markers^132^. In another family, three QTLs (on LGs 8, 15, and 16) were identified for carbon isotope discrimination (Δ^13^C), which is a proxy for seasonal WUE. These QTLs were stable across two years in a “Honeycrisp” × “Qinguan” family, and the LG 8 QTL co-localized with one of the previously mentioned QTLs for transpiration rate^133^. The WGS allowed the identification of 28 QTL-associated candidate genes and differentially expressed transcription factors between the high and low WUE cultivars “Qinguan” and “Honeycrisp”, respectively^134^, under drought stress^133^. Zhou and co-workers^135^ identified and characterized the *MdAGO* gene family coding for Argonaute proteins in apple and found them to be induced by drought, salt, cold, and ABA treatment, and *MdAGO4.1* was upregulated in the WUE cultivar “Qinguan” during water stress.

Other genes have also been connected to WUE by using apple genomics resources. FK506-binding proteins (FKBPs) play diverse roles in numerous critical processes for plant growth, development, and abiotic stress responses. In apple, 42 putative members of the FKBP gene family were identified across 13 chromosomes by performing BLAST analysis of 23 FKBP proteins of *Arabidopsis* against predicted apple proteins^136^. An Arabidopsis ROF1/ROF2-mediated network of 11 other interactive proteins was generated, and their apple homologs were identified on the apple WGS. Subsequent qRT-PCR analysis indicated that, under water-deficit or NaCl treatments, 10 gene pairs were co-expressed and showed uniform upregulation, indicating that genes within this apple MdFKBP62a:MdFKBP65a/b-mediated network have potentially important roles in water-deficit and NaCl-stress signaling^136^, making them possible targets in breeding for high WUE. RNA binding proteins play important roles in plant responses to biotic and abiotic stresses, including for apple. The YT521-B homology (YTH) domain-containing RNA binding protein (YTP) was first found in *Rattus norvegicus* and is related to oxygen-deficient stress^137^. A WGS survey led to the identification of 26 putative YTP gene models in apple^138^. Next, *MhYTP1* and *MhYTP2*, characterized in *M. hupehensis*, were implicated in drought response after 6 days and differentially expressed in several plant tissues with and without stress^137^ and overexpression of this gene enhanced WUE in transgenic plants^139,140^.

Nutrient uptake and transport efficiency (NUTE) in apple are genetically complex traits specific to each nutrient and involving both root-specific (referring to the rootstock in commercial production conditions) and scion-specific components^141,142^. NUTE is influenced by a wide range of physiological and morphological mechanisms, such as interaction with soil biota, active, and passive transport, vessel composition and size, root induced hormone concentration^124,143^, and interaction with pH, as well as management options such as crop load and irrigation^144^. Classical QTL mapping revealed QTLs influencing scion leaf concentrations for thirteen minerals^145^. Several of these QTLs have been validated in other experiments and in field applications (G. Fazio, manuscript in preparation). The WGSs and subsequently developed tools such as the 20K SNP array were instrumental to the fine mapping and anchoring of these QTLs as well as to the identification of several candidate genes through gene expression experiments (G. Fazio, manuscript in preparation).

The challenge in the next few years is to develop WGSs that are more relevant and representative of apple germplasm involved root and rootstock traits. More than 750 million trees in the ground worldwide are grafted on “Malling 9” apple rootstock, which is more than any scion cultivar planted, yet except for a few Illumina paired end sequencing efforts no assembled complete rootstock WGS is available. Rootstock cultivars often include species other than *M*. *domestica* in their pedigree, such as those derived from “Robusta 5” (*Malus* ***×*** *robusta*), the source of several valuable rootstock attributes including resistance to fire blight and apple canker^146^ and improved nutrient uptake. There are opportunities in obtaining genome sequences and developing efficient genome-scanning tools that represent such species to advance rootstock research.

## Tree dormancy genes and genetics: awakening well-known candidate genes

Temperate zone fruit trees, including apple, modulate their growth rhythm to adapt to seasonal environmental changes such as temperature and day length. Such trees use bud dormancy to adapt to winter cold. Bud dormancy can be fundamentally defined as the inability of the meristem to resume growth under favorable conditions. Following exposure to a genetically determined specific chilling period, known as chilling requirement (CR) fulfillment, and a subsequent warming period, known as heat requirement (HR) fulfillment, dormant buds can shift to the bud break stage. CR and HR fulfillment associated with winter and spring temperature changes, respectively, are believed to be important for the achievement of uniform flowering in commercial orchards. Global climate change affects chilling accumulation during winter, resulting in the occurrence of abnormal bud dormancy phenology, including morphological bud disorders, bud burst delay, and low bud burst rate. Although current breeding programs mainly focus on improvement of yield, fruit quality, and disease resistance, additional objectives such as climate change adaptation should receive increased attention in the near future. Characterization of genes and genetic mechanisms underlying bud dormancy, CR, HR, bud break, and flowering time is therefore relevant for future apple breeding.

Recent transcriptomic studies using information from the apple WGS has shed light on the possible crucial roles for dormancy regulation in apple of candidate genes for two key transcription factors, *DORMANCY-ASSOCIATED MADS-box* (*DAM*) and *FLOWERING LOCUS C-like* (*FLC-like*)^147–152^. There is also evidence of metabolite dynamics correlated with many cold-related and dehydration-related gene expression changes during dormancy. Both lipids and raffinose family oligosaccharides accumulated in buds during dormancy, and their biosynthetic and modification pathway-related gene expression changes coincided with dormancy phase transitions^153,154^. These metabolites might play roles in carbon storage and signal transduction for establishment of dormancy. Strikingly, carbon-limiting conditions and carbon starvation responses underlie bud dormancy in woody and herbaceous species^155^. Collectively, WGS information has enabled research that has provided new insights into the biochemical pathways underlying dormancy.

The study of flowering time in several apple bi-parental and multi-parental populations revealed a major QTL on LG 9 and secondary QTLs on LGs 8, 12, and 15 depending on the genetic background^156–158^. Candidate genes were identified close to these QTL intervals, especially *DAM*s and *FLC-like* homologous genes^158^. Genomic information from the GDDH13 WGS and the high-density Axiom® Apple480K SNP array enabled a powerful GWAS of flowering time in apple, which confirmed the involvement of LG 9 in the genetic control of flowering time^84^. Information from the WGS has also facilitated epigenetic studies such as DNA methylation analysis of winter dormancy^159^. The use of such approaches in the future will also help clarify the possibility that epigenetic memory is coupled with dormancy regulation triggered by environmental signals.

## Flowering genes: new knowledge blooming from the genome

Combining the apple WGS with analyses of conservation or divergence of flowering genes offers important clues as to the environmental and physiological factors that influence flowering in apple. Many well-studied flowering genes and gene families in model plant species—including *FT*, *TFL1*, *SOC1*, *SPL*, *LFY*, and *AP1*—are conserved in the apple genome. This observation suggests that the basic genetic wiring controlling flowering, as characterized in other eudicots, can be used as a blueprint for dissecting the genetics of flowering in apple. In contrast, apple has no clear counterparts of many other genes known to have more specialized functions in other plants. For example, apple appears to lack a counterpart of *FLC*, a MADS-domain transcription factor from *Arabidopsis* that acts as a major repressor of flowering in the absence of a vernalizing cold period. This absence is consistent with the observation that apple flowers are initiated in mid-summer, independently of cold temperatures.

The relatively recent duplication of the apple genome created two copies of most genes, offering a rich context in which to further explore the diversification of gene function and elaborations on the basic mechanism of flowering in apple. The *FT* gene is an interesting example. In *Arabidopsis*, the protein product of the *FT* gene is produced in the phloem tissues of the leaf and trafficked along the translocation stream to the shoot apical meristem, where it promotes transcription of *AP1* and other genes that direct flower formation^160–162^. However, in apple, the duplicated *FT* genes *MdFT1* and *MdFT2* are expressed mainly outside the leaf, in distinct patterns, with *MdFT1* expressed in the apical meristem during floral induction and *MdFT2* in the floral structures and young fruit^163,164^ (Gottschalk and van Nocker, unpublished results). This situation suggests that *FT* might mediate promotion of flowering independent of photoperiod, consistent with the apparent day-neutral flowering habit of apple.

The apple WGS will also assist in understanding the effects of plant growth regulators on flowering in apple, potentially leading to development of novel compounds that could increase commercial production efficiency. For example, the phytohormone gibberellic acid (GA) has long been known to repress flowering in apple, in contrast to its well-known promotion of flowering in rosette herbaceous plants^165,166^. It was recently reported that exogenous GA applied to apple trees early in the growing season was associated with increased expression of *MdTFL1-1*, one of two copies of the *TFL1* gene, late in the growing season, at a time when flowers would be forming^167^. *MdTFL1* can repress flowering when expressed ectopically in *Arabidopsis* and apple^168^, and, if *MdTFL1* does act as a flowering repressor in apple, then its promotion by GA would provide a simple explanation for the repression of flowering by this phytohormone. Other genes such as *TEMPRANILLO* (*TEM*), *FLORALTRANSITION AT MERISTEM* (*FTM1*) and *SQUAMOSA PROMOTER BINDING PROTEIN-LIKE* (*SPL*) could also operate as flowering repressors, as suggested by an approach combining QTL detection and transcriptome analysis^169,170^.

The reference WGS will also provide insights into the genetic mechanisms underlying juvenility—arguably the most significant challenge for rapid cultivar development^171^. Indexing the full complement of apple genes, together with the finding that some well-studied mechanisms of phase change in maize and *Arabidopsis* are conserved in woody perennial plants^172^, provides a smooth route for developing apple breeding germplasm to enable the rapid introduction of novel phenotypes in response to rapidly changing production environments and market demands. In addition, an improved genomic knowledge of apple will shed light onto the interesting variance in juvenile period length observed among *Malus* wild and cultivated accessions. The rapid-cycling, wild apple species *M. hupehensis* has been used as a physiological, and more recently molecular, model to study juvenility^173^. However, unlike most *M. domestica* cultivars and other *Malus* species, *M. hupehensis* is triploid, and its allopolyploid genome presents significant technical challenges for sequence assembly. The high-quality *M. domestica* genome sequence will provide a roadmap towards understanding the genetic basis for variance in this trait in *M. hupehensis*, and ultimately lead to faster and more efficient breeding of new apple cultivars.

## Fruit ripening genes and genetics: maturing research

The apple WGS has been a fundamental resource for the determination of key genes involved in the climacteric response and cell wall modification of apple fruit. The attractiveness of apple fruit is due to an array of features (shape, size, color, texture, and aroma), which from the initial phase of fruit development continuously changes through the fruit ripening process^174^. In climacteric fruit, including apple, fruit ripening is triggered and coordinated by the action of the hormone ethylene. This simple gaseous hydrocarbon is synthesized through the Yang’s cycle that, starting from methionine, produces ethylene through the action of three main enzymes: S-adenosyl-l-methionine synthase (SAMS), 1-aminocyclopropane-1-carboxylic acid synthase (ACS), and 1-aminocyclopropane-1-carboxylic acid oxidase (ACO)^175,176^. Following synthesis, this hormone is perceived by a series of receptors (ethylene response sensor and ethylene resistant) that initiate a downstream signaling mitogen-activated protein kinase cascade (constitutive triple response—ethylene insensitive 2) activating finally a series of ethylene-dependent responses mediated by ethylene response factor transcription factors^177,178^. The ethylene competitor 1-methylcyclopropene (1-MCP) has commonly been used to reveal the role of ethylene in controlling the overall ripening process in apple. Investigation of the genome-wide transcriptional signature was enabled by use of two microarrays (iRIPE and WGAA) designed using the information made available by the apple WGS. Through use of these tools, a gene de-repression or de novo activation, was revealed following 1-MCP treatment^179^. Gene annotation and prediction using the apple WGS enabled detection of possible cross-talk between ethylene and auxin. Specifically, interference at the ethylene receptor level stimulated expression of *Aux*/*IAA* and *AUXIN RESPONSE FACTOR* (*ARF*) elements involved in the auxin signaling pathway^179,180^. Upregulation of these genes was further validated through a RNA-seq survey performed to investigate the physiological mechanisms controlling apple superficial scald^181^. Gene annotation also facilitated the detection of important elements involved in postharvest ripening, as well as in the control of this scald. Furthermore, the apple WGS enabled development of a new microarray (AryANE v1.0)^182^ that was used to investigate the cell wall modification process during ripening. This research identified a specific pectin methylesterase (*MdPME2*) as well as elements involved in hemicellulose metabolism^183,184^.

The apple WGS has also facilitated identification of QTLs associated with control of fruit quality. Genome re-sequencing of 14 apple accessions^11^ enabled prediction of eSNPs, which were subsequently included in the 20K SNP array. With this SNP array, QTLs associated with mechanical and acoustic components of apple texture were detected via PBA^81^. The same tool enabled a GWAS that revealed the genetic relationship between texture and the production of volatile organic compounds^185^. Here, the apple WGS facilitated anchoring of the QTL intervals and cataloging the underlying gene sets.

Further improvement of the quality of the genome assembly and fine-tuning of the gene annotation should enable more precise and informative characterization of the relevant genes involved in the control of important physiological pathways underlying the apple fruit ripening processes, ultimately enabling the development of new cultivars characterized by superior fruit quality.

## Fruit color genes and genetics: beyond skin depth with the *MYB10* gene

Fruit appearance is a key factor in consumer acceptance^186^. Red coloration in the skin and flesh of apple fruit has been demonstrated to be controlled genetically (by allelic variation)^187–190^, by the environment (differences in light and temperature)^191,192^ as well as by management (e.g., bagging vs. not bagging fruit). Fruit color is determined by anthocyanin accumulation in the vacuole (mostly cyanidin-3O-galactoside), which is regulated by the MYB1/MYB10 transcription factor in combination with bHLH and a WD40 partners in a protein complex^92^. *MYB1* and *MYB10*, which are likely to be allelic^192^, regulate the activity of the genes of the anthocyanin pathway and their expression correlates with anthocyanin accumulation. A locus closely linked to *MYB10* has been mapped and associated with red flesh and red skin using biparental populations and GWAS in germplasm collections^13,83,189,193–195^. Interestingly, this red fruit (*R*_f_) locus is orthologous with red color-imparting loci detected in other Rosaceae fruit crops such as cherry, peach and strawberry^196^. Recently, a robust allele-specific qPCR marker was developed and validated using both apple breeding families and genetically diverse cultivars^197^. Despite the relative simple genetic control of fruit color (the *MYB10* locus has been demonstrated to consistently explain greater than 80% of the phenotypic variance for fruit skin color in populations varying from no red overcolor to much overcolor), the underlying causative variant of this phenotypic variation has not yet been identified. However, the causative variant for red flesh coloration compared to the usual white flesh color has been identified. “Type 1” red flesh has been demonstrated to be due to a repeated motif in the regulatory region upstream of the *MYB10* open reading frame^190^.

The availability of the new GDDH13 reference genome^8^, together with whole genome re-sequencing data for a range of apple cultivars (old and modern)^10,57^, opens the prospect of pinpointing the causative variant(s) for red skin color. The GDDH13 WGS assembly offers a unique “haplotype 0” opportunity, as “Golden Delicious” is homozygous for the non-red skin allele. DNA variants located in regulatory motifs upstream of *MYB10* are obvious candidates to be examined first, as they might alter *MYB10*’s activity and consequently influence the downstream activity of the anthocyanin biosynthesis genes. For example, some evidence of epigenetic modification in the promoter region upstream of *MYB10* has been demonstrated in striped “Honeycrisp” apples^198^. More re-sequencing, including using bisulfite-treated DNA, might shed new light on the key regulatory motifs. Such work will need to be complemented with a test of the efficiency of contrasting-effect haplotypes for anthocyanin gene activation, activation of *MYB10* itself, and as targets of transcription factors regulating *MYB10*. One key consideration is that red fruit skin might be associated with more than one allele. In this case, a combination of PBA using a population segregating for red coloration derived from several ancestral sources of red determinism combined with high-throughput genome re-sequencing would be required.

## Fruit acidity genes and genetics: epistatic allelic variation in a regulatory pathway

Malate accounts for more than 90% of total fruit organic acids in apple determined by concentration^199^. Fruit malate content varies widely in *Malus* accessions: from 0.5 to 22.7 mg/g, with 2.2 times more malate detected in wild species than in *M. domestica* cultivars^200^. Enzymes and their coding genes associated with malate biosynthesis and degradation were previously emphasized, until the important role was uncovered of malate transport into the vacuole and its maintenance there^201–203^. Major differences in apple fruit acidity among cultivars were determined to be controlled by a single locus that was mapped to one end of chromosome 16 and designated as the *Malic acid* (*Ma*) locus^102,204,205^. Expression of *Ma1* co-segregated with malate content and at least one allele sufficed for increased *Ma1* expression, causing a three-fold increase in fruit malate content^206^.

Exploration of the apple WGS enabled major discoveries after more than a decade of stagnation in characterizing the actual gene underlying apple fruit acidity^111,207,208^. The *Ma* locus, explaining 17.0–42.3% of phenotypic variance in fruit pH and titratable acid, was narrowed down to a genomic interval no larger than 150 kb containing 44 genes. Next, the interval was narrowed down further to 65–82 kb containing 12–19 genes. Finally, a G-to-A mutation was identified at 1455 bp of the open reading frame in the *Ma1* gene that leads to a premature stop codon, truncating 84 amino acids of an aluminum-activated malate transporter (MdALMTII) protein, and significantly reducing fruit acidity when occurring as a homozygote (*mama*). However, this natural occurring G-to-A SNP in the coding region of *MdALMTII* could not fully explain observed variation in fruit acidity among diverse *Malus* accessions or during fruit developmental changes. This is not surprising, as other acidity QTLs have been reported which occasionally had a size similar to *Ma1*. A QTL on LG 8 exhibited a similar or higher contribution to fruit acidity in many other segregating families when compared with the LG 16 QTL^29,102,209,210^, although also a substantial lower contribution has been reported^205^. This difference in performance might be due to different functional alleles or tightly linked QTLs of different effect segregating among families^211^. Enabled by whole genome re-sequencing, genetic variation in three genes on chromosome 8, *MdSAUR37*, *MdPP2CH* and *MdMYB44*, were identified and validated. The regulatory pathway of *MdSAUR37*, *MdPP2CH*, and *MdALMTII* was dissected and appears to fully explain the hierarchical epistatic genetic control of apple fruit acidity^212^.

The expression levels of 3066 genes exhibited significant correlation with malate concentrations during fruit development in “Golden Delicious”^213^. More than 1300 genes were differentially expressed between cultivars with *MaMa*/*Mama* versus *mama* genotypes^214^. Several vacuole transporters, V-ATPase, MdVHA-A, MdVHA-B1, MdVHA-E, MdVHP1, MdALMT1, MdALMT6, MdALMT9, and others, some of which under the direct regulation of MYB transcription factors or MdCIPK24-like proteins, were determined to play a pivotal role in determining malate content^215,216^.

Future research targets utilizing the apple WGS include development of a more detailed network of apple fruit malate metabolism and discovery of further natural genetic variation in the network in diverse *Malus* germplasm resources. Emphasis should be on the molecular regulation pathways of malate transport to the vacuole, vacuole malate sequestration and depletion during fruit development and post-harvest storage, the effect of different growing environments and functional mutations among cultivars and species. One potential challenge is that in some cultivars acetic acid and tartaric acid can account for 10–31% of total acids^210^, which together with various classes of aromatics and sugars create the colorful flavors of apples.

## Disease resistance genes and genetics: straight to candidate R-genes

The availability of the apple WGS has revolutionized the approaches and reduced the time needed to identify candidate resistance genes. Just over 20 years ago, the mapping of a resistance gene (*R* gene), which is the first step towards the positional cloning of a gene, was a long and tedious endeavor. Genetic markers such as random amplification of polymorphic DNA (RAPD), amplified fragment length polymorphism (AFLP), and restriction fragment length polymorphism (RFLP) were used, generally in combination with bulked segregant analysis (BSA)^217^, to (roughly) map the *R* genes. These genetic markers then had to be transformed into SCAR or cleaved amplified polymorphic sequence (CAPS) markers to be useful for marker-assisted selection and/or to start the cloning of an *R* gene. Around the beginning of the new millennium, genetically mapped SSR markers for apple became available^204,218–220^. In a very short time period, these markers allowed mapping of a series of *R* genes, such as the apple scab resistance genes *Rvi2* and *Rvi4*^221^, *Rvi5*^222^, *Rvi11*^223^, *Rvi12*^224^, and *Rvi15*^225^, as well as the fire blight resistance genes *FB_E* and *FB_Mf*^226^ and *FB_MR5*^227,228^, mildew resistance genes^229,230^, and several QTLs for resistances (e.g., see ref. ^231–233^).

The establishment of genetic map positions of these resistance genes opened up their potential for cloning and determination of their DNA sequence. Prior to the availability of the first apple WGS of Velasco and co-workers^1^, “workarounds” were necessary to identify markers closely associated with a gene of interest. For example, during the positional cloning of *Rvi15*, Galli and co-authors^234^ developed tightly linked markers using the sequence of a “Florina” bacterial artificial chromosome (BAC) clone of the homolog region. Parravicini and co-authors^235^ performed an AFLP-BSA approach to clone the fire blight resistance gene *FB_E*. To our knowledge, Fahrentrapp and co-authors^236^ were the first to report the use of the apple WGS to fine-map an *R* gene in apple, *FB_MR5*. In this case, the apple WGS allowed a rapid saturation with markers of the regions containing the *R* gene. The time savings can also be recognized by the increase in number of published reports of such research. Before the release of the apple WGS, entire papers were published reporting “only” the fine mapping of an *R* gene (e.g., see ref. ^234,237^). In contrast, after the genome release, such work was summarized in relatively short sections of publications, and papers reported not only the fine mapping but also the identification of candidate genes, such as for apple scab (*Rvi1, Rvi 5, Rvi 12*, and *Rvi18*)^238–241^ and fire blight (*FB_MR5*, *FB_Mfu10*^228,236,242^). The WGS also facilitated research on genetic variation for resistance genes, such as the identification of *FB_MR5* analogs in other wild apple species^243^. Finally, the WGS facilitated determination of the diversity and identity of QTLs from different studies for the same linkage group by clarifying their relative chromosomal position^244^.

However, identification of many candidate *R*-gene allele(s) cannot be achieved using the currently available apple WGS, because these resources have not been developed from individuals in the germplasm that carry the resistance alleles. Until now, *R*-gene alleles have been identified by sequencing BAC clones from libraries of resistance allele-carrying individuals spanning the *R*-gene regions (e.g., see ref. ^234–236^). The fast progress in sequencing technology and sequence assembly associated with decreasing sequencing cost will soon make the use of BAC libraries obsolete. Instead, whole genome resequencing of individuals carrying an *R* allele of interest and the subsequent assembly of the specific region carrying that *R* allele is expected to become the new standard. Furthermore, determining the correct order of repetitive genes will be very important, because resistance genes are often arranged in tandem repeats^245^.

With the WGS of apple speeding up the process of identifying *R* genes, translation to crop improvement is also increasing. Genetic markers and knowledge gained from *R*-gene cloning research is allowing the building of pyramids of *R* alleles against the same pathogen with different mechanisms of resistance. Such pyramids of *R* alleles are expected to lead to the development of cultivars with more durable resistances. These alleles could be combined into single individuals by traditional crossing leading to new apple cultivars, or alternatively be introduced into popular cultivars by cisgenic or CRISPR/Cas9 approaches in countries where this is permitted. Protocols in apple for the introduction by CRISPR/Cas9 of large genetic regions (e.g., entire *R* alleles) are beginning to be reported^246,247^. However, for some diseases a more straightforward approach might be the targeted knock-out of susceptibility *S* genes, such as for *MdMLO19*^248^ that should lead to development of powdery mildew resistant apple cultivars, at least in the absence of other *MLO* genes of similar effect^249^.

## Trait-predictive DNA test development: monitoring major genetic factors for breeders

Rapid advances in apple breeding were projected in the publications of both the first and second iterations of the apple WGS^1,8^. Indeed, progress is being made in the development and application of genomic technologies in the area of trait-predictive tests, although perhaps not as quickly as hoped for by earlier reviewers^171,250^. The use of SNP arrays^10–12^ and GBS to efficiently screen mapping populations and construct relatively dense genetic maps^13,251^ has made the mapping of trait loci much less challenging and correspondingly the number of QTLs in the Genome Database for Rosaceae^252^ has risen rapidly since the availability of the apple WGS. The recent development of a tool utilizing apple resequencing data from breeding germplasm, for the purpose of identifying SNPs that are unique to the accession carrying an attribute of interest, has both increased the efficiency of genetic mapping of trait loci and provided a source of SNPs for use in designing transferable high-throughput markers^253^ in combination with accurate pedigree records or PBA^74,75^. Highly reliable diagnostic markers derived from candidate genes for traits can now be readily developed by examining the DNA sequence at MTLs such as major resistance loci and the sequence underlying QTLs^254,255^.

Early adopters of trait-predictive tests mainly employed simple sequence repeat and SCAR markers^256–260^ and highly informative SSR markers are still used for tracing pedigrees^67,69^. Currently, high-throughput SNP-based markers have become the first choice for use in trait-predictive tests for parental and seedling selection by breeders and many programs have moved to develop and introduce the use of such tests^80,254,260–263^. As marker-assisted selection (MAS) was introduced into the apple breeding program at the New Zealand Institute for Plant & Food Research Limited (PFR) before the advent of the apple WGS^257^, PFR researchers across several disciplines were in a sound position to exploit the new knowledge to increase efficiencies and speed up progress towards the PFR goal of multi-resistant new cultivars with pyramided alleles across multiple loci for durable resistance. The PFR scion breeding program employs diagnostic SNP markers to combine alleles at MTLs for resistance to apple scab. Currently, in order to be cost-effective, a phenotypic screen for *Rvi2* is followed by MAS for *Rvi6*. In some families, MAS is applied for additional quality traits, such as red flesh (*Myb110a*). Both breeding parents and stage 2 selections are routinely screened with a range of markers including those for resistances to scab (*Vh8*/*Rvi8* and *Vr*/*Rvi19*), powdery mildew (*Pl2*), fire blight (*Fb-R5*), European canker (*Rnd1*), and woolly apple aphid (*Er1*, *Er2*, *Er3*). The PFR apple rootstock breeding program has employed MAS since 2012, to reduce the population for detailed phenotyping for time-consuming horticultural traits to less than 10% of the initial numbers^253,264^. Screens applied vary according to year and genetic background of seedlings, but include dwarfing (*Dw1*, *Dw2*) resistances to fire blight (*FB-R5*), European canker (*Rnd1*), and woolly apple aphid (*Er1*, *Er2*, *Er3*), as well as adventitious rooting^265^. Nearly all diagnostic markers are applied in the cost-efficient fluorescent probe-based Taqman® format, with more than 25,000 seedlings screened annually in the combined PFR apple breeding programs as a tool to breed better cultivars faster. MAS targeting trait loci for fruit quality and disease resistance is also in routine use in several other apple breeding programs around the world^266^.

MAS can be and is also routinely employed to reduce numbers of seedlings prior to evaluation by genome-wide selection^171^. Other types of high-throughput markers have been developed for apple, e.g., KASP™ in the European project FruitBreedomics^261^ and there are now commercial services for high-throughput marker screening in most areas of the world.

Various avenues of future work are warranted to facilitate the application of trait-predictive tests for MAS. Individual breeding programs will wish to develop markers for specialized traits that are relevant to their own germplasm and breeding goals. New marker options need to be investigated for apple, e.g., GT-Seq, which generates genotypes by NGS from relatively small panels of targeted SNPs^267^. Further work is required to develop user-friendly bioinformatic tools for access and sharing of sequence data, as well as comprehensive pipelines for the development of specific marker classes, from NGS through to primer/probe sets. Additional development of haplotyping, already initiated with SNP arrays, could be made using re-sequencing and tools such as Beagle^268^.

## Genomic prediction: genome-wide performance forecasting, black box, and beyond

The technology of genomic prediction (GP, also known as genomic selection, genome-wide selection/prediction, and whole genome prediction) was developed in animal genetics to target traits influenced by more than a few large-effect QTLs and soon used widely in animal, forestry and crop improvement^269–271^. Apple was the first horticultural tree crop in which this approach was explored^194,272^ and implemented^269^. The premise of GP is that anonymous genome-wide markers are sufficiently dense so that most or all QTLs are in LD with a marker and that most or all QTLs segregate with the use of multi-parent populations. Training populations of multiple families are used to develop prediction models by simultaneously fitting all markers as random effects with a prior distribution^273^ in contrast to conventional marker-assisted selection which uses only significant large-effect QTLs detected through linkage analysis or GWAS for selection/prediction.

Prediction accuracy, the correlation between true genetic effect and phenotype predicted only from markers in a validation population, is a major driver of GP success but varies greatly (−0.02 to 0.89) across several apple studies^194,272,274,275^. Consistently, GP accuracy has been demonstrated to be higher than using large-effect QTL-only models for polygenic traits (reviewed by Crossa and co-workers^276^). Results from apple studies suggest important factors leading to higher accuracy are close genetic relationships between training and validation populations^194,274^, large training population sizes^194,275^, small effective size of training and validation populations^194,274^, high heritability of traits particularly obtained through objective phenotyping^194,272^, assessment of continuously distributed traits^272^, and high density of markers^272^. However, multiple assessments across years of the same experimental unit (i.e., pseudo-replication^277^) might not efficiently improve heritability and hence not improve accuracy^272^. The extent of LD in a population is related to effective population size, hence LD and genetic relationship levels are entangled in a population, making it difficult to separate their effects on the accuracy of GP. Genomic relationships among training population individuals can reliably be estimated with a few thousand SNPs^278^ but many QTLs might not be in strong LD with such a low density of SNPs due to the distribution of SNP and QTL locations across the genome. LD between SNPs and QTLs, and relatedness between training and selection candidates, would decay over generations^83^. Hence, the accuracy of GP over successive generations would reduce unless GP models are recalibrated, although the equivalent is also needed with any linkage-based prediction method^279^. To date, population sizes are considerably smaller, and density of genotyping lower, in apple studies compared to those for animals.

The influence of using different prediction models has been explored in apple. Assumption of a Gaussian distribution (i.e., many markers with small effect or approximate quantitative trait model) has been the most common in apple GP studies^194,274,280^, but other distributions are possible^281^ and have been used^272^ although no effect was found on prediction accuracy^194,282^. Genomic prediction models can combine whole-genome prediction and detection of large-effect QTLs^283–285^ and in apple a Bayesian LASSO model was used to detect regions of high influence for fruit quality traits^194^. The Gaussian framework, however, allows extension to more complex models^276^ and has been used to model both additive and non-additive genetic effects, although negligible effect on prediction accuracy of breeding value (additive) or clonal values was reported in apple^274^ and cherry^286^. These models were also used to study genotype-by-environment interaction (G×E) for fruit quality traits^274,287^.

The fundamental aim of GP is greater gain at reduced cost and in less time than conventional strategies^276^ and considerable increases in gain across animals and crops has been demonstrated^271^. Use of GP for parental breeding value selection and juvenile clonal value selection was reported in The New Zealand Institute for Plant & Food Research Limited (PFR) apple scion breeding program to bypass the initial stage of juvenile testing by reducing the initial population by 90%^269^. However, as many traits are of interest in apple^288^, this strategy might work best when focusing on a single trait that is governed by many loci of minor effect. Selection of parents from the training population provides a strong genetic link with the selection population, to improve prediction accuracy. GP is reportedly in initial use in US apple programs (J. Luby, Uni. Minnesota, pers. comm.).

As GP is a unifying technology across animal and plant breeding^289^, advances across these systems can provide direction for fundamental discoveries and practical application in apple. The ability to clone apple individuals provides opportunities to counteract low prediction accuracy due to small sample sizes or large experimental variation. Improvement in accuracy from multi-environment and multi-trait prediction models found in other crops^276,290,291^ needs to be verified for apple. Inclusion of biological information, such as previously identified large-effect QTLs and/or extension to crop growth (development) or functional structural models might also help increase prediction accuracy^271,275,292,293^. The biallelic nature of SNPs suggests combining linked SNPs into haplotype blocks (haploblocks) is likely to be more informative of the variability at a QTL and could decrease hidden variability, hence leading to higher prediction accuracy^271,294,295^. While the use of sequence information to build GP models suggests higher accuracy because the causal variation should be directly included in the model, the value of this extension in apple needs to be verified as populations are highly structured^69^ such that large haploblocks could be efficiently tagged with a lower density of markers^271,296^. Fast-track breeding via transgenesis^297,298^ or induced through a plant virus vector^299^ might enable full realization of GP potential in apple. While SSRs were used to select for a favorable QTL allele and against unfavorable genetic background over five generations in seven years^300^, GP can be used to identify elite progeny across a range of traits. Implementation of GP for apple improvement might require modification of breeding programs and stochastic simulation can be used to evaluate benefits of different strategies^290^.

The ability of GP to connect otherwise unconnected populations without the need for clonal replication^287,301^ provides the foundation for combining data from small local breeding programs to improve accuracy, dissect G×E, predict genetic potential across a global environment, and use as a reference for other research. This approach can therefore leverage the latent value of historically collected trial data. The RosBREED project assembled a large set of apple phenotypic and genotypic data from across 14 global locations (three USA, seven EU, two New Zealand, and two Australian) to develop a global GP model for fruit sweetness and acidity and evaluate the accuracy of predictions. Bioinformatics tools are being developed to manage and deliver the technology so that the performance of new germplasm can then be routinely predicted.

## Epigenetics: scratching the surface

Access to a near-complete apple WGS provides the foundation for epigenetic studies. A key area unlocked by the availability of the high-quality GDDH13 WGS is the study of sports (i.e., clones showing a novel phenotype) and, associated with this, the study of epigenetic mechanisms and their contribution to important traits such as fruit size and color. By definition, epigenetic changes result in heritable changes in gene expression that cannot be explained by changes in the DNA sequence. To be able to study true epigenetic events, one has to ensure that there are as few genetic changes as possible in the genome under examination. Therefore, apple sports are therefore an excellent starting point to study epigenetics in plants. Although some sports might be the result of genetic changes (such as mutations induced by transposable elements^302^, others might vary only at the epigenetic level and more specifically at the DNA methylation level.

The main reason for generating the apple reference genome GDDH13 was to enable an understanding of the epigenetic mechanisms involved in regulating fruit size. This approach was successful, as the research generated a list of genes likely involved in fruit size regulation^8^. Currently, there is no direct impact of apple epigenetics research on crop improvement. Future studies using epigenetic markers on sports and on larger populations will enable assessment of the contribution of DNA methylation to traits of interest. Considering that DNA methylation also influences fruit color intensity and patterning in apple sports^198,303^, it is likely that we have only started to scratch the surface of the contribution of epigenetics to traits of economic importance.

Several epigenetic studies are expected to profit from the availability of high-quality apple WGSs in the near future. The study of the genetic and epigenetic mechanisms influencing phenotypic changes of apple sports is straightforward with a good genome assembly and will allow the rapid identification of genes involved in important agronomic traits, including fruit color and shape, disease resistance and tree architecture. Chemical-induced or targeted changes in DNA methylation patterns could be used to produce novel traits of interest by gene demethylation. DNA methylation changes can also be used to induce the artificial mobilization of transposable elements^304^, which results in a powerful tool for gene discovery (via tagged mutations) and the generation of phenotypic diversity.

## Genome database for Rosaceae: the researcher’s toolbox

Apple researchers exploiting the apple WGS are well provisioned with data and tools housed in the Genome Database for Rosaceae (GDR). The GDR^305^ (https://www.rosaceae.org) is the publicly accessible, central repository and data-mining resource for genomics, genetics, and breeding data of the Rosaceae family, which includes apple as well as other economically important crops. The GDR is built using Tripal^306,307^, a resource-efficient, open-source platform for online biological database construction. Prior to the availability of the first WGS in 2012, the GDR contained mostly EST data, genetic markers, and genetic maps. The new data types currently included in the GDR include multiple WGSs, reference transcriptomes, QTLs, SNP array data, and phenotypic and genotypic data. Increased data from the worldwide apple research community, combined with active curation, analyses, and tool development, has resulted in significant expansion of the GDR during the last few years. Described below are the currently available data and interfaces, with a focus on new features.

The physical structure of the apple WGS, as described by its nucleotide sequence, is the foundation of apple genomic resources in the GDR. Genomic data include the GDDH13 WGS^8^ along with three historical versions of the heterozygous apple genome assembly^1^. Although no chromosomal sequence is available for these heterozygous apple genome assemblies, the sequences of overlapping contigs are available, which in turn have been aligned to the chromosome using markers from the genetic map. For the Golden Delicious v1.0 WGS, however, a set of four pseudo-haplotype assemblies (primary and alternatives 1, 2, and 3) with chromosomal sequences, derived from the contigs of the original v1.0 assembly, is available for download. The primary pseudo-haplotype data is also available in the Search Genes page and JBrowse^308^ in the GDR. Researchers can readily examine in fine detail how the apple genome compares physically with those of other crops. The most recent apple WGS is being used in synteny analysis with eight other Rosaceae whole genome assemblies using MCScanX^309^, with the results available through the new Synteny Viewer.

Another strong focus of the GDR is apple gene function. Since 2017, the community database has provided an apple reference transcriptome (*M. domestica* GDR RefTrans V1) that combines published RNA-Seq and EST data that are computationally annotated for homology to genes of other plant species and assignment of InterPro domains^310^ and GO terms^311,312^. The data can be accessed in various ways: through the Species page, the Search Genes and Transcripts page, the JBrowse^308^ tool, or BLASTX^313^ tool.

Genetic linkage, marker and trait locus information needs are well served. The GDR contains 108 genetic maps for *Malus*, which can be viewed and compared through a new graphic interface, MapViewer. Currently, the GDR contains 2.6 million genetic *Malus* markers, of which 99.7% are SNPs while more than 7000 are other marker types. SNP data include those of the 9K^10^, 20K^11^, and 480K^12^ apple arrays. Most of the remaining SNPs are those identified in silico from accession resequencing conducted during development of those SNP arrays. The SNP data are available as JBrowse tracks, downloadable files, and from the Marker Search page. The Marker Search page has a new Filter-by-Trait name feature, which enables researchers to find markers associated with QTLs. In addition, the SSR and SNP genotype search pages contain data from ten genotyping projects. Trait locus data in the GDR includes 1528 QTLs and 36 MTLs for 143 horticultural traits of apple. Although impressive, some caution is necessary in considering these numbers. They are based on the accumulation of QTLs as independently reported in the literature and therefore might include redundancies across studies or treatments (including years) within a single study. In addition, some caution is be needed in using reported QTL information. Their assignment might arise from different levels of statistical significance; regrettably, it is often not specified whether a genome-wide or a chromosome-wide threshold was applied. Some reported QTLs were from an interval mapping procedure without subsequent co-factor analysis. Such QTLs have increased risk of being spurious or being assigned to an incorrect position, while other true QTLs might remain hidden^314,315^.

Phenotypic data for breeding germplasm are also accessible on the GDR, from publicly funded projects such as RosBREED^78^. In addition to the Search Trait Evaluation page, the public breeding data can be accessed using the Breeding Information Management System (BIMS). BIMS allows breeders to store, manage, archive, and analyze their private or public breeding data.

The data of the original apple WGS^1^ enabled the GDR to integrate various data types. Functional annotation of gene models using sequence similarity and synteny analyses provided a first step in data integration across species. Alignment of transcripts and markers, used in genotyping and QTL mapping, to the WGS, enables researchers to utilize data of different types originating from various species and disciplines. Future targets include integrating new types of data such as pan-genome data, epigenome data, and phenomics data, as well as data standardization and further tool development. In this way, the GDR will continue to enable researchers to open new frontiers in understanding the structure and function of the apple genome and its components.

## Conclusion

Over the past decade, apple WGSs have had an enormous impact on our understanding of apple biological functioning, trait physiology and inheritance, leading to practical applications for improving this highly valued crop. The first apple WGS reported in 2010^1^, v1.0, was valuable as the initial reference for sequence information, fine mapping, gene discovery, variant discovery, and tool development. As the research community exploited this resource, the need for improvements in the genome assembly became increasingly apparent. The new GDDH13 assembly reported in 2017^8^ provided many of those improvements, and research leaps ensued. In contrast to a decade ago, causal gene identities for phenotypes of fundamental or practical interest can today be discovered rapidly. Genome-wide polymorphisms are readily screened for hundreds to thousands of germplasm individuals. Genetic maps are constructed readily and QTLs are quickly related to candidate genes and/or converted into diagnostic tests for breeding use. We now understand the species, geographical, and genomic origins of domesticated apple more precisely, as well as the crop’s relationship to potentially valuable sources of alleles in wild relatives. Availability of the WGS is not only turbo-charging the conduct of these classical research steps to crop improvement, but is also driving innovative methods of achieving more durable, environmentally sound, productive, and consumer-desirable apple crop production.

Research collaboration on apple genomics and genetics, already vibrant across an international community, has much synergistic value. The physical map of apple’s 17 chromosomes is a shared foundation that unites diverse fields of apple science. Although it can be viewed in many ways, each chromosome has the same physical entity for any researcher. The apple WGS with its set number of chromosomes and their lengths delineates the boundaries of what has arisen through natural means to coalesce as the “blueprint” of an apple tree. Researchers worldwide are resolutely identifying the roles and influences, intra-genomic interactions, and germplasm distributions of variants of chromosomal modules—the genes, motifs, trait loci, haploblocks, base pairs and so on—all using the WGS of apple. Integration of the apple transcriptome, proteome, metabolome, epigenome, QTLome^316^, and so on, across experiments and experimental material will surely expand our scientific understanding of apple. The sheer amount of multi-dimensional genomic and downstream data generated has elevated the need and opportunities for their coordination to a new level. To sustain such collaborative advances, the GDR now serves as the research hub for the international apple genomics community. We should also think beyond apple: exploiting the genomic synteny of apple with crop relatives in the Rosaceae family has only just begun, and holds much potential for wider collaboration and research breakthroughs.

Advances are needed in upgrades to the base genome sequence data pool. The reference WGS should be improved to ensure its completeness and accuracy. A first step could be noting in genome browsers any suspicious regions identified by comparisons between the WGS and high-quality genetic linkage maps. Given the possibility that the doubled-haploid individual underlying the current reference WGS is missing parts of its genome compared to typical heterozygous cultivars^8^, there is still a need for a reference WGS representing the complete cultivated apple genome. In any case, the linear or “vertical” representation of the apple WGS needs to be extended laterally to capture sequence variation along each chromosome. Development of the apple pan-genome would be a clear way forward for describing the current allelic variation of cultivated apple. Expansion of the pan-genome to encompass the *Malus* genus is a further compelling target as *M. domestica* is able to be crossed with dozens of other species.

Advances are also needed in use of WGS-related data to answer fundamental and applied research questions. There is an urgent need for an apple gene atlas describing the roles and relationships of apple’s 42,140 genes, as currently more than half the putative protein-coding genes of apple have unknown functions. Another challenge is understanding the interactions of genes with the external world, be they via transient methylation, inherited epigenetic changes, or statistically trackable and exploitable interactions of G×E (×M, crop management, and ×A, age or ontogeny of plants). Genomic prediction approaches promise a foundation for genotype and environment matching. A major leap in analytical capabilities is required to gain information on the vast reservoir of alleles that already contribute to the phenotypes of cultivated and wild apple trees. For apple WGS information to be able to directly support new cultivar development, user-friendly bioinformatics solutions are needed. Tools are required for ready conversion of trait-predictive DNA tests to commonly used and new genotyping platforms and for whole-genome-profiling diagnostics. Streamlined data curation workflows would enable any given breeding program to benefit, by translating genotypic data into genetic information that supports breeding decisions. Intuitive analytical tools are needed to track, over generations, all alleles of interest linked along each chromosome. Ultimately, such tools will facilitate understanding of genetic relationships across germplasm and empower “Breeding by Design”^317^ for breeders to access allelic diversity in available germplasm and design and target whole-genome ideotypes built from alleles of interest.

The curious apple scientist thirsts for knowledge—for every question answered, many more are raised. The apple WGSs have already enabled the answering of numerous questions. Tangible impacts have resulted because of the integration of apple genomics and genetics research with breeding. We hope the examples of apple WGS impacts inspire creative new ideas that spur further advances in understanding and improving apple.
